# Large scale hydrogeochemical and isotopic observations in the Baltic Sea system

**DOI:** 10.1038/s41597-025-06217-9

**Published:** 2025-11-06

**Authors:** Tristan McKenzie, Claudia Majtényi-Hill, Linnea Henriksson, Wilma Ljungberg, Gloria M. S. Reithmaier, Luiz C. Cotovicz, Jannine M. Lencina-Avila, Nico Mitschke, Michael Ernst Böttcher, Beata Szymczycha, Adam Ulfsbo, Aprajita S. Tomer, Tibaud Cardis, Per O. J. Hall, Ceylena Holloway, Júlia Rodriguez-Puig, Solveig Börjesson, Yvonne Y. Y. Yau, Shibin Zhao, Henry L. S. Cheung, Stefano Bonaglia, Tobia Politi, Linda Zetterholm, Nicolai Verbücheln, Iris Schmiedinger, Gregor Rehder, Thorsten Dittmar, Mats Tysklind, Pedro A. Inostroza, Ana Tronholm, Isaac R. Santos

**Affiliations:** 1 Department of Marine Sciences, University of Gothenburg, Gothenburg, Sweden; 2 Centro de Estudos do Mar, Universidade Federal do Paraná, Pontal do Paraná, PR Brazil; 3 Department of Marine Chemistry, Leibniz Institute for Baltic Sea Research Warnemünde, Rostock, Germany; 4 Institute for Chemistry and Biology of the Marine Environment (ICBM), School of Mathematics and Science, Carl von Ossietzky Universität Oldenburg, Oldenburg, Germany; 5 Geochemistry & Isotope Biogeochemistry Group, Leibniz Institute for Baltic Sea Research, (IOW), Warnemünde, Germany; 6 Marine Geochemistry, University of Greifswald, Greifswald, Germany; 7 Interdisciplinary Faculty, University of Rostock, Rostock, Germany; 8 Marine Chemistry and Biochemistry Department, Institute of Oceanology Polish Academy of Sciences, Sopot, Poland; 9 National Marine Science Centre, Southern Cross University, Coffs Harbour, Australia; 10 Institut de Ciència i Tecnologia Ambientals, Universitat Autònoma de Barcelona, Bellaterra, Spain; 11 Departament de Física, Universitat Autònoma de Barcelona, Bellaterra, Spain; 12 Department of Geological Sciences, Stockholm University, Stockholm, Sweden; 13 Frontiers Science Center for Deep Ocean Multispheres and Earth System, and Key Laboratory of Marine Chemistry Theory and Technology, Ministry of Education, Ocean University of China, Qingdao, China; 14 Department of Chemistry, Umeå University, Umeå, Sweden; 15 Helmholtz Institute for Functional Marine Biodiversity (HIFMB) at the Carl von Ossietzky Universität Oldenburg, Oldenburg, Germany; 16 Institute for Environmental Research, RWTH Aachen University, Aachen, Germany; 17 Department of Biological and Environmental Sciences, University of Gothenburg, Gothenburg, Sweden

**Keywords:** Marine chemistry, Carbon cycle, Marine chemistry, Hydrology, Element cycles

## Abstract

We report hydrogeochemical and isotopic observations across the Baltic Sea from two research expeditions: (1) a ~5000 km cruise-track onboard the *R/V Skagerak* in 2023 and (2) a land-based sampling for terrestrial endmembers in 2024. The ship-based observations include continuous monitoring of hydrographic parameters, pH, and ^222^Rn in surface water. In addition, we collected 542 discrete samples from the water column, vertical profiles (n = 69 stations), and meteorological data. Land observations include discrete samples from beach groundwater (n = 77), nearshore surface water (n = 47), and rivers close to the coastline (n = 46). Discrete samples were analyzed for short-lived radium isotopes, nutrients, dissolved organic and inorganic carbon, total dissolved nitrogen, total alkalinity, methane, and stable isotopes (δ^18^O_H2O_, δ^2^H_H2O_, δ^13^C_DIC_, δ^13^C_CO2_, δ^13^C_CH4_). Data products include seven open-access files. This dataset forms the deposit for upcoming original research publications. This dataset will also be valuable to researchers interested in the hydrogeochemistry of coastal seas, like the Baltic Sea, and more generally interested in submarine groundwater discharge and estuarine biogeochemistry.

## Background & Summary

Coastal systems act as dynamic and vital reactors modulating the flux of water and elements between land and sea^1,2^. The biogeochemical functioning of the coastal ocean is supported by a balance of inputs from rivers, submarine groundwater discharge (SGD), sediments, and the atmosphere as well as outputs from water mixing with other water bodies and burial in sediments^1,3,4^. While these inputs and outputs maintain natural biogeochemical functioning, increased land-based and atmospheric chemical loads deliver excess nutrients, carbon, metals, and greenhouse gases to the coast^1,5,6^. SGD, for instance, frequently carries higher concentrations of nutrients and dissolved inorganic carbon (DIC) to the coast compared to rivers^3^, potentially driving eutrophication and coastal acidification^7,8^. Understanding the sources and transport pathways for water and dissolved chemical species and carbon species is critical for addressing pervasive water quality problems, eutrophication, and hypoxia in aquatic systems^9^.

The Baltic Sea is a large semi-enclosed marginal sea characterized by strong physiochemical gradients, particularly in salinity, dissolved oxygen, and associated element loads^10^. A restricted water exchange with the North Sea and substantial freshwater inputs result in a practical salinity gradient ranging from ~2 in the northernmost waters of the Bothnian Bay to 34 in the Skagerrak Sea^10,11^, creating estuarine conditions over ~2000 km. The limited water exchange also leads to long water residence times (~20–30 years in the surface layer)^12,13^. Water exchange in the deeper parts of the Baltic Sea is not only restricted laterally, but especially vertically due to a strong permanent halocline ~40–80 m below the surface that maintains hypoxic/anoxic conditions in large bottom areas^14^. The specific hydrography, deep basins supporting the development of euxinic conditions, and substantial anthropogenic impact has resulted in prolonged water quality issues such as eutrophication and hypoxia leading to significant changes in biogeochemical cycling^14–16^.

The Baltic Sea is a well-studied water body due to these long-term water quality problems^10,14,15^. Existing monitoring programs usually focus on the main sources (e.g., riverine nutrients) and implications (e.g., oxygen levels, fish status) of eutrophication^10^. However, monitoring programs rely on repeat observations at the same stations to the detriment of large-scale snapshots of the entire region. In addition, groundwater tracer data (e.g., radium isotopes, ^222^Rn)^17–20^ have only been reported in a few local-scale studies in the Baltic region^21–26^. SGD has been identified as a critical, yet largely unquantified contributor to Baltic Sea water and biogeochemical budgets^27^. Here, we report results from extensive hydrogeochemical and isotopic characterizations collected during two Baltic-wide research expeditions. Our observations characterize groundwater and river waters and associated biogeochemical parameters in the Baltic Sea water column. These types of data are not only sparse in the Baltic Sea, but also in low oxygen environments in general. Therefore, these data contribute to Baltic Sea research and pollution modeling efforts as well as the growing community interested in SGD research.

This dataset is currently used by the authors for specific evaluations that will result in other scientific publications. The first of these publications quantifies vertical mixing and benthic silicate fluxes using vertical profiles of radium and silicate^28^, and estimates horizontal nutrient fluxes using ^224^Ra observations in coastal transects^29^. The full dataset is of further potential to those interested in topics such as coastal hydrology (e.g., SGD), marine biogeochemistry, and benthic-pelagic coupling. The data may also be of interest to those working with coastal seas like the Baltic Sea, as well as for comparative studies with other regions (e.g., North Sea, Black Sea, Mediterranean Sea) or on the global scale.

## Methods

### Sampling approach

**Fig. 1:**
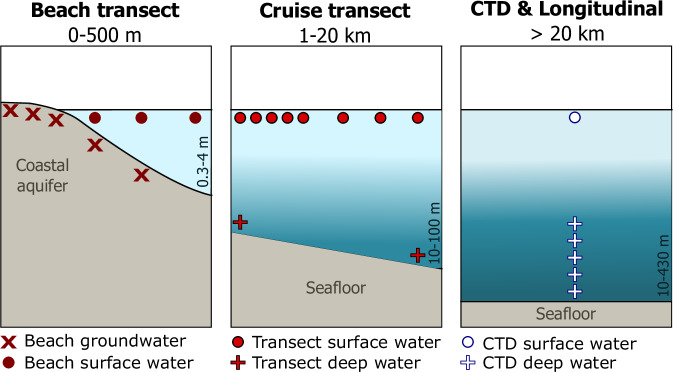
Schematic of site and sample types. Beach transect sites included beach groundwater 0.5 m below the sediment surface (dark red X’s) and nearshore surface water (dark red circles) up to 500 m from the shoreline sampled in 2024. Cruise shore-perpendicular transects aligned with beach transects (red circles) were sampled 1–20 km from the shoreline in 2023. For cruise transects, deep water (red crosses) was sampled at the transect endpoints. Conductivity, temperature, depth (CTD) sites included deep water vertical column samples (white crosses) and surface water samples (white circles). Additional sample types include longitudinal sites (white circles, surface water only sites during the cruise) collected between the CTD sites and river surface water (land sampling campaign, see Fig. 2B).

We collected hydrogeochemical data during two extensive Baltic Sea expeditions targeting both marine surface and deep waters, as well as land-based endmembers, including river and beach groundwater (Fig. 1).

**Fig. 2:**
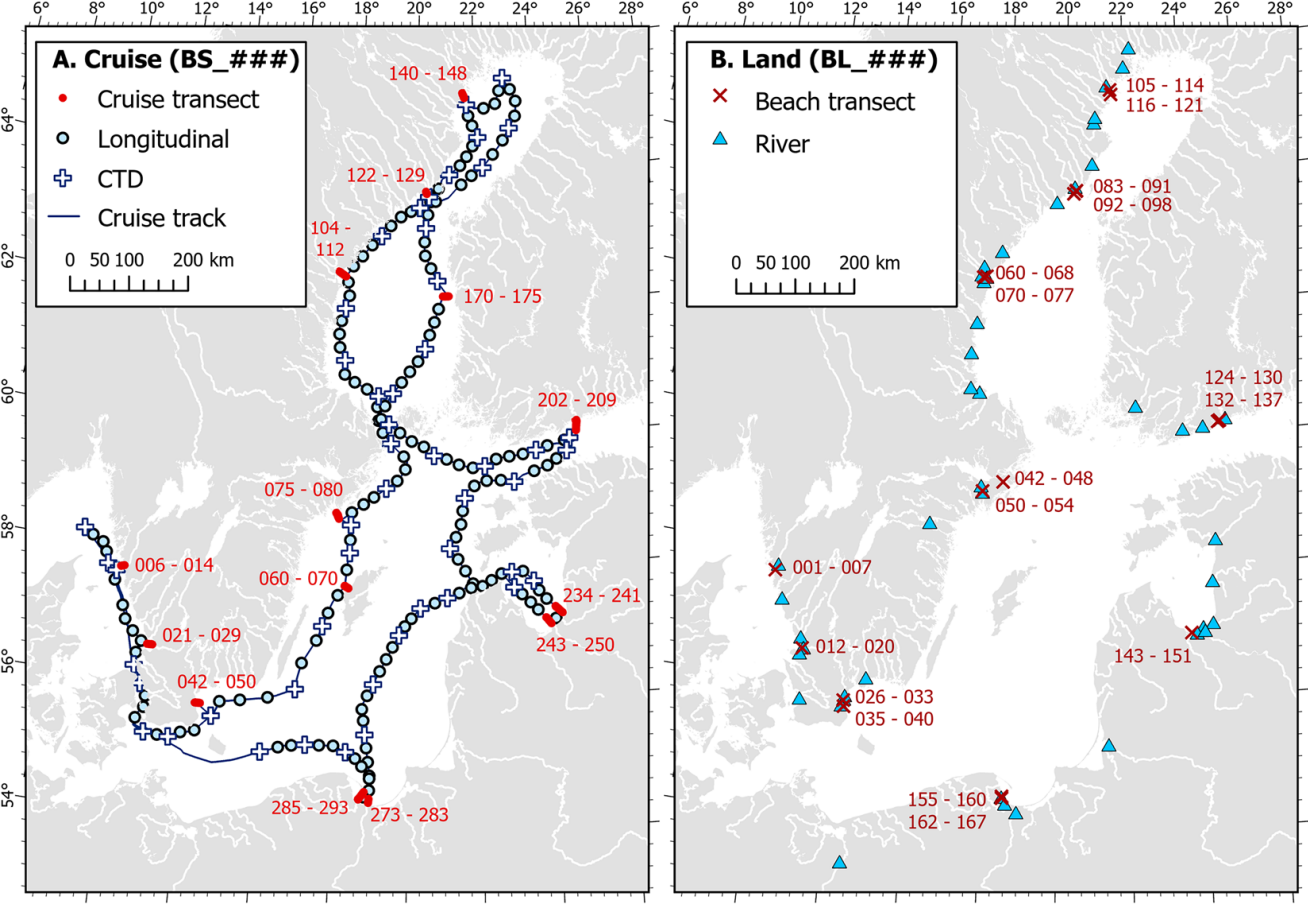
Map of sample locations. (**A**) Cruise track (blue line) and 302 discrete sampling stations included in the cruise data. Cruise station numbers (BS_###) progress from 001–303 (only transect stations are labelled in the figure for readability). CTD stations are indicated by white crosses. Longitudinal stations are indicated by light blue circles. Stations belonging to cross-slope transects are indicated by red circles with the range in station numbers in red text. (**B**) Land-based sampling, with sample numbers BL_### ranging from 001–170. Beach transect sites (including nearshore surface water and groundwater samples) are shown by dark red X’s, with corresponding sample numbers labeled in dark red text. Blue triangles denote river sampling stations Fig. 2B).

Sampling of the Baltic Sea water column was conducted onboard *R/V Skagerak* from September 10 through October 3, 2023. We collected spatial surface water survey data, vertical water column depth profiles via conductivity, temperature, depth (CTD) instrument casts, and discrete water samples along a 5,000 km cruise track (Fig. 2A). Overall, 542 discrete samples from 302 stations were collected, including 302 samples from surface water (~4 m depth) and 240 samples from the deeper water column from CTD casts. The sampling scheme for discrete samples included three main types of stations – CTD, longitudinal, and transect. Among these main station types, sample types included surface and deep water (Table 1). Longitudinal stations were spaced on transects every ~20 km, where only surface water samples (n = 134) were collected. CTD stations were spaced approximately every 4 stations and consisted of deeper water (n = 217) and surface water (n = 50) samples. Cruise transects (n = 14 transects) consisted of shore-perpendicular transects where we collected both surface water (n = 118) and deep water (n = 23) samples. The approach for sampling transects included a surface and deep water sample pair at the closest and furthest from shore stations (~10 to 20 km apart), with typically 7 surface water stations in between these two points at ~1–5 km intervals (higher density closer to shore). Date, time, duration of sampling, and station/sample type were noted for all stations and samples.

**Table 1:** Number of discrete samples collected by station type and from surface water, deep water, and groundwater.

Station Type	Surface water	Deep Water	Groundwater	Total Samples
**Cruise Longitudinal**	134	—	—	134
**Cruise CTD**	50	217	—	267
**Cruise Transect**	118	23	—	141
**Beach Transect**	47	—	77	124
**River**	46	—	—	46

A second, land-based sampling campaign targeting terrestrial endmembers took place in May and August 2024 (Fig. 2B). During this campaign, a total of 170 discrete samples were collected from rivers (n = 46), beach groundwater (n = 77), and beach surface water (n = 47) to characterize the multiple terrestrial endmembers discharging into the Baltic Sea. Beach samples were organized along shore-perpendicular transects and targeted the same areas as the cruise transect sites (Figs. 1, 2B). Surface water samples (rivers and beach surface water) were collected from the upper 0.5 m of the water column. Groundwater was sampled by digging holes on the beach until the water table was reached. We then used a peristaltic pump to fully drain the standing water in the hole three times, allowing the hole to fully recharge before finally taking the sample. Samples were then collected using a peristaltic pump. While we carefully sampled recently-recharged beach groundwater, groundwater sampling may be subject to minor degassing that could lead to underestimated concentrations of CH_4_, CO_2_, and DIC^30^.

### Cruise sampling campaign

#### Surface water survey sampling

We conducted a continuous underway spatial surface water (~4 m depth) survey for hydrographic parameters. An onboard Ferrybox system^31^ (Ferrybox I; -4H-JENA engineering GmbH, Germany) was used to measure water temperature (°C), specific conductivity (S/m), and practical salinity with a SBE 45 sensor at 1-minute intervals. Additional variables, including datetime (Coordinated Universal Time; UTC), latitude (decimal degrees N), longitude (decimal degrees E), course (degrees), ship speed (nautical miles per hour), oxygen concentration (mg/L), oxygen saturation (%), chlorophyll-*a* (µg/L), phycocyanin (ppb), turbidity (Nephelometric Turbidity Unit; NTU), and pressure (mbar) were logged simultaneously on the Ferrybox system. Salinity refers to practical salinity following the standard established by the Joint Panel on Oceanographic Tables and Standards^32^, unless otherwise specified. pH (total scale) in surface water was measured using a CONTROS HydroFIA pH flow-through pH analyzer (measurement interval = every 10 minutes). During the measurements, the dye m-Cresol Purple is added to the sample, and the absorption spectra are measured using VIS absorption spectrometry at 25 °C, allowing for calculation of pH^33^. The HydroFIA pH has an analytical accuracy of ± 0.01 pH units and precision of 0.005 pH units^34,35^.

#### CTD sampling

Vertical depth profiles and discrete deep water samples were collected from 69 stations (including 19 transect stations) via the onboard CTD (Sea-Bird SBE 911, SBE 43, WET labs ECO AFL/FL) and rosette water sampler (Sea-Bird SBE 32). At each CTD station we collected six discrete deep water samples, in increments starting ~2–4 m from the seafloor (labelled “BOT”), and then 10, 20, 30, 40, and 50 m from the “BOT” sample as well as a surface water sample. In some cases, fewer samples were collected due to a shallower water column. Measured parameters include datetime (UTC), pressure (dbar), water depth (m), water temperature (°C, Sea-Bird SBE 3), conductivity (m/S, Sea-Bird SBE 4), salinity, dissolved oxygen concentration (mL/L, Sea-Bird SBE 43), dissolved oxygen saturation (%, Sea-Bird SBE 43), fluorescence (mg/m^3^, WET Labs ECO-AFL/FL), light transmission (%), and turbidity (NTU, WET Labs ECO).

#### Meteorology

Continuous monitoring of meteorological parameters was obtained with an onboard weather station (Observator OMC) and logged every 2 minutes during the cruise. An average was taken in cases where two measurements were logged at the same time stamp at slightly different latitudes or longitudes ( ± 0.000003°). Parameters collected include datetime (UTC), air temperature (°C, OIC-406 probe, ± 0.1 °C accuracy), relative humidity (%, OIC-406 probe, ± 0.8% accuracy), air pressure (mbar, OMC-506 sensor, ± 0.3 mbar accuracy), relative wind speed (m/s, OMC-160, ± 2% accuracy), and wind direction (degrees, OMC-160, ± 3° accuracy).

#### ^222^Rn

A ^222^Rn spatial surface water survey was conducted throughout the cruise. ^222^Rn activities (Bq/m^3^) in surface water were measured using a radon-in-air detector (Durridge RAD7) with RAD-AQUA accessory^36^. Water was run continuously from the onboard pump system to the air-water exchanger and then to the radon-in-air detector with a measurement interval of 30 minutes. The minimum detection limit of the instrument for ^222^Rn in air was 4.0 Bq/m^3^. Overall, the mean analytical uncertainty during the survey (2σ; given as an output from the RAD7 instrument) was 63% for data points above the minimum detection limit. RAD7 uncertainties are based on counting statistics and integration time, thus higher uncertainties were due to relatively low ^222^Rn activities for most of the survey and short integration time (30 min). Considering only data points with ^222^Rn in water concentrations >30 Bq/m^3^, the mean analytical uncertainty becomes 34%.

#### Land sampling campaign water parameters

During the land sampling campaign, standard water quality parameters including water temperature (°C; ± 0.2 °C accuracy), salinity ( ± 1% accuracy), pH (NBS scale; ± 0.2 units accuracy), and oxygen saturation (%, ± 0.1% accuracy) were measured using a calibrated YSI ProDSS Digital Water Quality Meter. Latitude and longitude were determined using a handheld GPS. For beach transect sites, distances between sampling points were measured using a measuring tape to ensure accuracy and to correct for GPS resolution.

### Experimental analyses for discrete samples

#### Short-lived radium isotopes

Radium samples were collected into 5 L (groundwater), 60 L (cruise deep water and land-based surface waters), and 300 L (cruise surface water) containers. The collected water was passed through cartridges containing 20 g of MnO_2_-coated fibers that extract radium. ^224^Ra (t_1/2 = _3.6 days) and ^223^Ra (t_1/2 = _11.4 days) activities were then measured with a Radium Delayed Coincidence Counter (RaDeCC)^19^ within 48 hours of sample collection. Samples were re-run 4 weeks after collection to correct ^224^Ra activities for ingrowth of its parent isotope, ^228^Th. Quality control of the raw radium data included screening for counting anomalies such as spurious counts and leaks. Further quality control on the raw radium counting data assessed for cross-channel interferences due to logistical constraints that did not allow for a second 1 week count to correct for ^227^Ac, which can impact ^223^Ra^37^.

Samples collected from low oxygen deep water (n = 94) were processed by aligning 2–4 cartridges containing MnO_2_-coated fibers to quantify a potentially lower radium extraction efficiency due to the presence of sulfides and lack of oxygen. These samples were then analyzed on the RaDeCC as described above. Radium activities from the low oxygen deep waters were calculated by quantifying the radium extraction efficiency^28^. All radium activities were corrected for radioactive decay to the sampling time. RaDeCC detector efficiency was quantified at both the beginning and end of each sampling campaign. Final uncertainties (median =  ± 6.1%) represent 2σ and account for detector efficiency, counting statistics, and radioactive decay^38^. Samples with fewer counts than the background or <0.02 cpm in the 220 channel were below the minimum detection limit.

#### Dissolved inorganic nutrients

Samples for dissolved inorganic nutrient concentrations (µmol/L) were first collected into 60 mL syringes, which were then passed through a 0.45 µm cellulose acetate filter into 50 and 15 mL polypropylene Falcon tubes. Dissolved inorganic nitrogen (DIN; sum of NO_3_^−^, NO_2_^−^, and NH_4_^+^) and dissolved inorganic phosphorus (DIP; PO_4_^3−^) samples were kept frozen until analysis. Dissolved silicate (DSi; SiO_4_^4−^) was kept at 4 °C until analysis. All dissolved nutrients were analyzed with an AA500 AutoAnalyzer (SEAL Analytical) at the Institute of Oceanology, Polish Academy of Sciences. Repeat measurements of certified reference materials (CRM RM-BU; National Metrology Institute of Japan) were used to confirm analytic quality Accuracy, expressed as recoveries, was >99% for all parameters, while precision, indicated by the relative standard deviation, was <2%. The detection limit was 0.006, 0.003, 0.045, 0.012, and 0.027 µmol/L for NO_x_ (NO_3_^−^ + NO_2_^−^), NO_2_^−^, NH_4_^+^, PO_4_^3−^, and SiO_4_^4−^, respectively. Final NO_3_^−^ concentrations were calculated by subtracting the NO_2_^−^ concentration from NO_x_.

#### Dissolved Inorganic Carbon and Total Alkalinity

Samples for dissolved inorganic carbon (DIC) were collected into 60 mL syringes and immediately filtered through 0.7 µm GF/F filters into 12 mL borosilicate glass exetainers without headspace and stored at 4 °C until analysis. Samples were not poisoned due to prohibited use of mercury(II) chloride (HgCl_2_) at Swedish institutions. Instead, DIC samples were analyzed within 24 hours (onboard the research vessel for the cruise sampling) and 72 hours (land sampling) of collection to mitigate sample alteration. DIC concentrations in filtered, unpoisoned samples can change by ±0.5 to 2% within 4 days of sample collection according to in-house lab storage experiments^39^. DIC samples were analyzed using an AS‐C5 (multi‐port version, Apollo SciTech, LLC) with a non-dispersive infrared CO_2_ detector (LI‐850, LI‐COR, USA) using 3% H_3_PO_4_ acid with 7% NaCl, and lab grade N_2_ carrier gas. Samples were run in replicates of 3–5 times, until three replicates consistently met a suitable precision (<±2 and ±1 µmol/kg for the samples measured during the cruise and land campaigns, respectively). Final DIC concentrations (µmol/L) were calibrated using certified reference materials^40^ (CRM Batch nos. 189 and 203) using the replicate mean and converted from µmol/L to µmol/kg using *in-situ* salinity and the temperature measured during the analysis.

The method for analyzing total alkalinity (TA) differed slightly between the cruise and land sampling campaigns. During the cruise sampling, we collected TA samples in 250 mL borosilicate glass bottles, which were then kept for ~20 min in a 20 °C water bath before analysis ( <24 hours of sample collection). TA was then measured via a semi-closed potentiometric titration technique using Gran-point evaluation with 0.05 M HCl^41^. The system measures alkalinity in µmol/L. During the land sampling, water for TA analysis was filtered through 0.7 µm GF/F filters into 50 mL polypropylene Falcon tubes. Samples were stored at 4 °C and analyzed with semi-closed potentiometric titration using Gran-point evaluation with a high precision titrator (Metrohm 888 Titrando with Tiamo light)^42^. Concentrations <2500 µmol/kg were titrated with 0.01 mol/L HCl, concentrations >2500 µmol/kg with 0.025 mol/L HCl. For both the cruise and land campaigns, TA samples were calibrated with certified reference material^40^ (CRM Batch nos. 189 and 203 for the cruise and CRM Batch no. 216 for the land). For all samples and CRM analyses, TA in µmol/kg was calculated using *in-situ* salinity and the temperature measured during the titration. The mean precision was <±3 µmol/kg and ±6.7 µmol/kg for the cruise and land sampling, respectively.

#### Methane concentrations

Samples for dissolved CH_4_ concentrations were sampled directly from the Niskin bottle into 60 mL syringes via gas tight tubing. Samples were then transferred into 22 mL borosilicate glass vials and spiked immediately with 0.2 mL 7 M ZnCl_2_ for preservation^43^. Vials were capped without headspace and stored at 4 °C until analysis. The sample was measured 4-5 months after sample collection using an N_2_ gas headspace technique^44^ with a flame ionization detector gas chromatography (Thermo Scientific Trace 1300). Two certified gas standards were used for calibration (Air Liquide Gas AB): one low concentration (1.9 ± 0.04 ppm), and one high concentration (50.1 ± 1.0 ppm). For samples with headspace concentrations <1.9 ppm, only the low concentration standard was used, with the calibration curve was forced through origin. The detection limits were ~0.2 ppm, with an analytical precision (expressed as relative standard deviation of multiple 1.9 ppm CH_4_ standard area counts; n = 50) of 2.8% and accuracy (represented by the relative error) of 2.2%. Dissolved CH_4_ concentrations (nM) were calculated from the measured headspace concentration (ppm) based on the solubility coefficient^45^. Replicate sample analyses resulted in a median precision of 8% for the peak area and 5% for the headspace concentration based on the relative standard deviation.

#### δ^13^C_DIC,_ δ^13^C_CH4_, and δ^13^C_CO2_

Samples for δ^13^C_DIC_ were collected into syringes and then passed through a 0.7 µm GF/F filter into borosilicate glass 12 mL exetainers. Cruise samples were kept frozen until analysis within 1.5 years of sampling. Samples from the land campaign were frozen until arrival at Leibniz Institute for Baltic Sea Research, where they were spiked with HgCl_2_. These samples were then kept under dark conditions at 4 °C until analysis within 10 months of sampling. Stable isotope analyses by means of continuous-flow isotope-ratio mass spectrometry (CF-irms) were carried out using a Thermo Finnigan MAT253 gas mass spectrometer attached to a Thermo Electron Gas Bench II via a Thermo Electron Conflo IV split interface^46^. Sample solutions were allowed to react with H_3_PO_4_ for at least 18 h at 23 °C in the Gasbench II before introduction into the gas mass spectrometer. International calibration materials (NBS-19, IAEA Li carbonate standard), and Solnhofener Plattenkalk were used to calibrate the measured isotope signals against the V-PDB scale. According to replicate measurements of standards, the reproducibility was better than ±0.1‰^46^.

Samples for δ^13^C_CH4_ and δ^13^C_CO2_ were collected directly into 60 mL syringes with gas tight tubing and transferred in 250 mL and 112 mL borosilicate glass bottles fixed with 0.4 mL 7 M ZnCl_2_^47,48^. Bottles were sealed with butyl rubber stoppers and stored without headspace in 4 °C. δ^13^C_CH4_ and δ^13^C_CO2_ were measured within 1 year of sampling using a Small Sample Isotope Module (SSIM Picarro) coupled to a cavity ring-down spectrometer (Picarro G2201-i) using synthetic air (HiQ Synthetic Air 5.0; Linde Gas) as carrier gas and headspace. Samples outside the instrument operation ranges of 1.5 to 50 ppm CH_4_ were diluted. The operation range for CO_2_ was 380 to 2000 ppm. Stable isotope standard gas (Airgas Specialty Gases) was used to calibrate stable isotope values (−69 to −45‰, and −29 to −8.6‰ vs. V-PDB for δ^13^C_CH4_ and δ^13^C_CO2_, respectively). Precision (expressed as standard deviation) ranged from ±0.14 to ±1.08‰ for δ^13^C_CH4_ and from ±0.60 to ±1.04‰ for δ^13^C_CO2_. Accuracy (expressed as absolute error) ranged from 0.96 to 7.30‰ and 0.56 to 4.09‰ for δ^13^C_CH4_ and δ^13^C_CO2_, respectively. Quality control measures included removing samples with CH_4_ headspace concentrations <1.5 ppm. Five samples in the top and bottom 10th percentiles of the distribution were removed.

#### Dissolved organic carbon and total dissolved nitrogen

During the cruise campaign, we collected samples for dissolved organic carbon (DOC) and total dissolved nitrogen (TDN) analysis from the surface and bottom water at CTD stations. Water samples were taken from three different Niskin bottles from the CTD rosette into pre-rinsed 20 L carboys (HDPE). Samples were sequentially filtered through pre-rinsed 1.0 µm Causapure (CPR-001-09-DOX, polypropylene, Infiltec, Germany) and 0.1 µm Causa-PES (CPS-S10-10-DOV-A, polyethersulfone, Infiltec) filter cartridges into pre-rinsed 10 L carboys. An aliquot (30 mL) was collected in HDPE vials and immediately acidified with diluted hydrochloric acid (∼8 mol/L) to a pH of 2 for the analysis of DOC and TDN.

During the land campaign, we collected water samples into syringes and then filtered through a 0.7 µm GF/F filters into pre-cleaned 60 mL amber borosilicate vials and preserved with 0.2 mL of 1 M H_3_PO_4_. All samples were stored at 4 °C until further analysis.

DOC and TDN were measured in triplicates by high-temperature catalytic oxidation using TOC-VCPH/CPN and TOC-LCPH/CPN Total Organic Carbon Analyzers (both Shimadzu Corp.) within 1-3 months after collection at the Institute for Chemistry and Biology of the Marine Environment in Germany. Each analytical replicate consisted of three to five injections, with peak area variability kept below 3%. Analytical accuracy and precision were validated using the deep seawater reference as provided by the Hansell Organic Biogeochemistry Lab (University of Miami, USA). Accuracy usually fell within the consensus range of the reference material but never deviated by more than 2 µmol/L. Precision was generally better than 5% except for one run with a precision of 6.2%. All reported values exceeded the limit of detection, which was determined as the mean of ultrapure water blank measurements plus 10 times the standard deviation.

#### Water isotopes

Samples δ^18^O_H2O_ and δ^2^H_H2O_ analysis were collected into 4 mL glass vials without headspace and kept cool and dark until measurement within 1.5 years and 7 months of sample collection for the cruise and land campaigns, respectively. Stable isotope analysis was carried out by means of Laser-cavity ring down spectroscopy (LCRDS) using a Picarro L2140-i^49^. For brackish water samples a metal liner was used to reduce the potential effect of the salt^50^. Reference materials (SLAP, VSMOW, USGS48, internal standards) were used to calibrate the measured isotope ratios towards the Vienna Standard Mean Ocean Water (VSMOW) scale. Results are reported in ‰ vs. VSMOW. Average analytical uncertainties for δ^18^O_H2O_ were ± 0.045‰ and ± 0.25‰ for δ^2^H_H2O_.

### Data processing

#### Initial quality control

We first performed conversions to ensure matching units for all data sources. All data time stamps were then converted to UTC. The survey and CTD data underwent additional processing and were matched to the discrete samples as described below.

#### Cruise surface water survey and discrete sample matching

Cruise surface water samples were first matched to the latitude and longitude logged on the Ferrybox based on sampling time and then confirmed with GPS notes taken by the ship captain during sampling. After this, other variables of interest from the Ferrybox (salinity, water temperature, dissolved oxygen saturation, and turbidity) were matched to discrete samples by taking the average value for the variable from the time sampling began until it was completed.

Survey data from the HydroFIA pH were matched to the Ferrybox data by date and time and then processed using the Ferrybox salinity data. Final pH values from the cruise are on the total scale at 25 °C and *in-situ* salinity. The conversion to 25 °C was made in PyCO2SYS v1.8.3^51^ on the total scale using the dissociation constants of carbonic acid^52^ and bisulfate^53^. A mean surface (<5 m) TA content (1538 µmol/kg) was used as the second carbonate system input variable. When converting to the total scale, the difference between using the mean surface TA value versus the closest discrete TA sample was 2.3 × 10^−5^ ± 5.0 × 10^−5^ pH units (n = 2568). This is at least two orders of magnitude lower than both the accuracy and precision of the method and does not introduce large systematic pH errors.

Post-processing and cleaning of ^222^Rn data included checking data quality for voltage spikes, that relative humidity remained below 10%, and time stamps converted to UTC. ^222^Rn survey data were matched to the Ferrybox data by downsampling the 1-minute interval data to 30-minute intervals using the mean and shifting by one time-step to account for equilibrium adjustment for the RAD7 instrument. ^222^Rn in exchanger air activities were then converted to ^222^Rn in water (Bq/m^3^ by calculating the Ostwald solubility coefficient^54^ using the matched water temperature and salinity data from the Ferrybox. Final ^222^Rn in water uncertainties were calculated using error propagation.

#### Cruise CTD profiles and discrete sample matching

Variables of interest from the raw downcast CTD profile data were extracted and processed from.cnv files using the Python package pycnv. Extracted CTD profiles for each station were cleaned, including removing rows that were flagged by the sensor for quality control reasons, and cutting the data at the maximum depth. Additional derived CTD variables including conservative temperature (CT; °C), absolute salinity (SA; g/kg), and water density (ρ; kg/m^3^) were calculated from CTD data using the Thermodynamic Equation of Seawater 2010 (TEOS-10) equation of state^55^ and the Gibbs Seawater (GSW) Oceanographic Toolbox in Python^56^. Discrete samples were then matched to CTD profile variables by water column depth.

## Data Record

Data products and the associated metadata can be found in an open-source Zenodo repository^57^. This dataset spans from September 10 to October 1, 2023, and from May 2 to August 28, 2024, for the cruise and land-based campaigns, respectively. Both campaigns cover latitudes 53.35 to 65.88 °N and longitudes 10.59 to 24.99 °E. In all, 35 variables were measured during the cruise and 22 variables during the land-based campaign. Uncertainties for all parameters in the dataset are either available on a per-sample basis (i.e., as an additional column in the dataset) or can be estimated on a per-method basis described in the text, allowing users to estimate values for their own work based on well-quantified uncertainty reporting. The data are organized into seven CSV files following Findable, Accessible, Interoperable, and Reproducible (FAIR) principles^58^:

1. Ferrybox surface water survey (1-minute resolution) (1_Cruise_Ferrybox_1min.csv)
2. Meteorological survey (2-minute resolution) (2_Cruise_meteo_2min.csv)
3. HydroFIA pH survey with matched and down-sampled Ferrybox data (10-minute resolution) (3_Cruise_HydroFIA_pH_10min.csv)
4. ^222^Rn surface water survey with matched and down-sampled Ferrybox and meteorological data (30-minute resolution) (4_Cruise_RAD7_30min.csv)
5. Processed CTD profiles (5_Cruise_CTD.csv)
6. Cruise campaign discrete sample data (6_Land_discrete.csv)
7. Land campaign discrete sample data (7_Land_discrete.csv)

Survey, CTD, and land-based discrete data variables, column names, and file names are summarized in Table 2. Discrete data can be found separate files for the cruise and land-based campaigns. For the land campaign, discrete measurements of 4 additional hydrographic parameters (salinity, water temperature, dissolved oxygen %, and pH) were also taken for each sample but are not shown in the Table 2.

**Table 2:** Summary of survey and vertical profile data collected on the cruise.

Variable	Source	Column Name	File name(s)
Datetime *UTC*	Surface survey (Δt = 1 min)	Datetime_UTC	1_Cruise_Ferrybox_1min.csv
Latitude *decimal °E*	Surface survey (Δt = 1 min)	Latitude	
Longitude *decimal °N*	Surface survey (Δt = 1 min)	Longitude	
Water temperature *°C*	Surface survey (Δt = 1 min)	FB_temperature_C	
Conductivity *m/S*	Surface survey (Δt = 1 min)	FB_conductivity_S_m	
Practical salinity	Surface survey (Δt = 1 min)	FB_salinity	
DO *%*	Surface survey (Δt = 1 min)	FB_oxygen_sat_per	
Chlorophyll*-a µg/L*	Surface survey (Δt = 1 min)	FB_chlorophyll_ug_L	
Turbidity *NTU*	Surface survey (Δt = 1 min)	FB_turbidity_NTU	
Pressure *dbar*	Surface survey (Δt = 1 min)	FB_pressure_dbar	
Phycocyanin *ppb*	Surface survey (Δt = 1 min)	FB_phycocyanin_ppb	
Air temperature *°C*	Meteorology (Δt = 2 min)	airtemp_C	2_Cruise_meteo_2min.csv
Air humidity *%*	Meteorology (Δt = 2 min)	airRH_per	
Barometric pressure *mbar*	Meteorology (Δt = 2 min)	airpressure_mbar	
Wind speed *m/s*	Meteorology (Δt = 2 min)	windspeed_m_s	
Wind direction *°*	Meteorology (Δt = 2 min)	winddir_deg	
pH *Total scale*	Surface survey (Δt = 10 min)	HydroFIA_pH_Totscale	3_Cruise_HydroFIA_pH_10min. csv
^222^Rn in water Bq/m^3^	Surface survey (Δt = 30 min)	Radon_water _Bq_m3	4_Cruise_RAD7_30min.csv
Pressure *dbar*	CTD depth profile	CTD_pressure_dbar	5_Cruise_CTD.csv
Water temperature *°C*	CTD depth profile	CTD_temperature_C	
Conductivity *S/m*	CTD depth profile	CTD_conductivity_S_m	
Fluorescence *mg/m*^3^	CTD depth profile	CTD_fluor_mg_m3	
Turbidity *NTU*	CTD depth profile	CTD_turbidity_NTU	
Water depth *m*	CTD depth profile	CTD_depth_m	
Practical salinity	CTD depth profile	CTD_salinity	
DO *%*	CTD depth profile	CTD_oxygen_sat_per	
Density kg/m^3^	CTD depth profile	CTD_rho_kg_m3	
^224^Ra *dpm/100 L*	RaDeCC	Ra224_dpm_100L	6_Cruise_discrete_samples.csv 7_Land_discrete_samples.csv
NO_3_^−^ *µmol/L*	AA500 AutoAnalyzer	NO3_umol_L	
NO_2_^−^ *µmol/L*	AA500 AutoAnalyzer	NO2_umol_L	
NH_4_^+^ *µmol/L*	AA500 AutoAnalyzer	NH4_umol_L	
PO_4_^3−^ *µmol/L*	AA500 AutoAnalyzer	PO4_umol_L	
DSi *µmol/L*	AA500 AutoAnalyzer	Si_umol_L	
DIC *µmol/kg*	AS‐C5 DIC Analyzer	DIC_umol_kg	
δ^13^C_DIC_ *‰ vs. VPDB*	Thermo Finnigan MAT253, Gasbench II	d13C_DIC_permil_VPDB	
TA *µmol/kg*	Semi-closed potentiometric titration; 888 Titrando	TA_umol_kg	
DOC *µmol/L*	Shimadzu TOC-VCPH/CPN & TOC-LCPH/CPN	DOC_umol_L	
TDN *µmol/L*	Shimadzu TOC-VCPH/CPN & TOC-LCPH/CPN	TDN_umol_L	
δ^18^O_H2O_ *‰ vs. VSMOW*	Picarro L2140-i	d18O_H2O_permil_VSMOW	
δ^2^H_H2O_ *‰ vs. VSMOW*	Picarro L2140-i	d2H_H2O_permil_VSMOW	
CH_4_ *nM*	Thermo Scientific Trace 1300	CH4_nmol_L	6_Cruise_discrete_samples.csv
δ^13^C_CH4_ ‰*vs. VPDB*	Picarro 2201-i	d13C_CH4_permil_VPDB	
δ^13^C_CO2 ‰_ ‰ *vs. VPDB*	Picarro 2201-i	d13C_CO2_permil_VPDB	

The type of data and location (file name and column name within the file) are listed for each variable. For discrete data, cruise surface water samples include longitudinal (LONG), CTD, and transect station types. Cruise deep water samples include CTD and Transect station types. Land-based and nearshore samples include beach groundwater (GW), beach surface water (SURF), and river samples. ^223^Ra is not reported due to the high percentage of samples not meeting criteria for cross-talk interferences. Stable isotope data are presented in ‰, which is equivalent to the new milliUrey (mUr)^59^.

## Technical Validation

CTD data underwent a two-stage cleaning procedure. First, we removed data that were flagged by the instrumentation (given a value of −9.99 × 10^−29^ in any column, n = 106,911), which left 258,082 data points remaining. This was most frequently found at the start of the record for an individual CTD profile due to instrumentation startup and equilibration. We then cleaned the profiles to ensure the CTD was in the water and included downcast data only by cutting the record at the maximum depth recorded. This resulted in further removal of 6,195 rows, with 251,887 rows remaining in the final CTD record from all profiles.

Cleaning of survey (surface water basic water quality parameters, meteorology, and ^222^Rn) data was minimal compared to the CTD data. However, several periods of missing data were identified (Table 3). In the cases of the Ferrybox, HydroFIA, and meteorological data, these were caused by either instrumentation error or deliberate pauses in recording due to harboring to avoid storms or allow for crew changes. For the ^222^Rn survey, the first day of data were removed due to high (>10%) relative humidity in the RAD7, which can cause inaccurate readings.

**Table 3:** Data gaps in survey data from the cruise.

Source	Start data gap (UTC)	End data gap (UTC)	Reason
Surface water survey - Ferrybox	Sept 12, 2023, 23:52	Sept. 13, 2023, 02:04	Instrumentation error
	Sept. 13, 2023, 19:55	Sept. 14, 2023, 10:40	Ship docked in harbor, instrumentation off
	Sept. 17, 2023, 01:57	Sept. 17, 2023, 13:20	Ship docked in harbor, instrumentation off
	Sept. 19, 2023, 06:18	Sept. 20, 2023, 13:40	Ship docked in harbor, instrumentation off
	Sept. 23, 2023, 05:08	Sept. 25, 2023, 13:39	Ship docked in harbor, instrumentation off
Surface water survey – HydroFIA	Sept. 10, 2023, 03:30	Sept. 11, 2023 11:07	Instrument turned off
	Sept. 13, 2023, 20:00	Sept. 14, 2023, 10:41	Ship docked in harbor, instrumentation off
	Sept. 17, 2023, 02:04	Sept. 17, 2023, 13:19	Ship docked in harbor, instrumentation off
	Sept. 19, 2023, 06:14	Sept. 20, 2023, 13:39	Ship docked in harbor, instrumentation off
	Sept. 23, 2023, 04:38	Sept. 25, 2023, 13:38	Ship docked in harbor, instrumentation off
Meteorology - Observator OMC	Sept. 28, 2023, 21:56	Oct. 01, 2023, 06:33	Instrumentation error
^222^Rn - RAD7	Sept 10, 2023, 03:30	Sept 11, 2023, 07:30	Relative humidity >10%

All experimental data were analyzed using well-established methods and checked using method specific quality control measures. Discrete sample matching quality control included confirming the station name, location, and sampling date and time. As an additional quality control check, we calculated the difference in depth between the discrete sample with the matched CTD profile scan to ensure this value never exceeded 5 cm. A second data validation was carried out to ensure the matched cruise surface survey and CTD data were consistent with individual discrete samples. Variables from different sources (i.e., CTD, Ferrybox, and experimental data) in discrete cruise samples were plotted against each other identify outliers and unexpected trends. Additionally, surface and deep water data were plotted by station for CTD stations for comparison. For the land campaign, which only consisted of discrete samples, the final quality control consisted of checking for anomalously high or low values and were evaluated for any unreasonable trends between variables.

## Data Availability

Data are available at 10.5281/zenodo.15792010.

## Code availability

No custom code was generated for this project.
